# Impact of DNA repair pathways on the cytotoxicity of piperlongumine in chicken DT40 cell-lines

**DOI:** 10.18632/genesandcancer.26

**Published:** 2014-07

**Authors:** Saki Okamoto, Takeo Narita, Hiroyuki Sasanuma, Shunichi Takeda, Shin-ichiro Masunaga, Tadayoshi Bessho, Keizo Tano

**Affiliations:** ^1^ Division of Radiation Life Science, Research Reactor Institute, Kyoto University, Kumatori, Osaka 590-0494, Japan; ^2^ Department of Radiation Genetics, Graduate School of Medicine, Kyoto University, Sakyo-Ku, Kyoto 606-8501, Japan; ^3^ Eppley Institute for Research in Cancer and Allied Diseases, University of Nebraska Medical Center, Omaha, Nebraska 68198, USA.

**Keywords:** BRCA1, BRCA2, piperlongumine, oxidative stress, homologous recombination, chemotherapy

## Abstract

Piperlongumine is a naturally-occurring small molecule with various biological activities. Recent studies demonstrate that piperlongumine selectively kills various types of transformed cells with minimal toxicity to non-transformed cells by inducing a high level of reactive oxygen species (ROS). ROS generates various types of DNA lesions, including base modifications and single strand breaks. In order to examine the contribution of ROS-induced DNA damage to the cytotoxicity by piperlongumine, various DNA repair-deficient chicken DT40 cell-lines with a single DNA repair gene deletion were tested for cellular sensitivity to piperlongumine. The results showed that cell lines defective in homologous recombination (HR) display hyper-sensitivity to piperlongumine, while other cell lines with a deficiency in non-homologous end joining (NHEJ), base excision repair (BER), nucleotide excision repair (NER), Fanconi anemia (FA) pathway, or translesion DNA synthesis (TLS) polymerases, show no sensitivity to piperlongumine. The results strongly implicate that double strand breaks (DSBs) generated by piperlongumine are major cytotoxic DNA lesions. Furthermore, a deletion of 53BP1 or Ku70 in the BRCA1-deficient cell line restored cellular resistance to piperlongumine. This strongly supports the idea that piperlongumine induces DSB- mediated cell death. Interestingly, piperlongumine makes the wild type DT40 cell line hypersensitive to a PARP-inhibitor, Olaparib. The results implicate that piperlongumine inhibits HR. Further analysis with cell-based HR assay and the kinetic study of Rad51 foci formation confirmed that piperlongumine suppresses HR activity. Altogether, we revealed novel mechanisms of piperlongumine-induced cytotoxicity.

## INTRODUCTION

Reactive oxygen species (ROS) are generated during regular metabolic reactions. It is well recognized that ROS are harmful to cells. ROS induce damage to important cellular components such as DNA, RNA, lipid, and protein [1, 2]. Various ROS detoxification mechanisms counteract excessive ROS to protect cells. Increasing evidence also demonstrates that ROS is necessary for several physiological responses, including differentiation, immunity, metabolic adaptation and autophagy [3-6]. Thus, a fine balance between the production of ROS and the detoxification of ROS needs to be maintained for proper cell growth. Improper regulation of ROS contributes to human pathology, including cancers and aging. Activated oncogene-derived cancers show signs of formation and accumulation of replication-associated DNA damage in the early stage of cancer development. Since activated oncogenes are known to induce ROS, ROS- induced DNA damage might be one of the sources of replication-associated DNA damage [7-9]. Cancer cells counteract the elevated level of ROSs by increasing anti- oxidation defenses [10]. These adaptations seem to be unique to cancer cells and might be required for cancer cell growth. Due to these double-edged sword features of ROS, both antioxidants and ROS-inducing chemicals have been tried as cancer chemotherapeutics. Recently, piperlongumine was identified through a cell-based, high- throughput screening to selectively kill various types of transformed cells without minimal cytotoxicity to non-transformed cells [11]. Piperlongumine is a biologically active, naturally-occurring compound from the Piper species, *Piperaceae*. It has been shown that piperlongumine has various biological activities, including anti-microbial, anti-inflammatory and anti-tumor activities [12]. Raj et al. [11] demonstrated that piperlongumine increases the level of ROS and apoptotic cell death selectively in cancer cells. In the same study, it was shown that the expression level of oxidative stress response enzymes, such as Glutathione S-transferases (GSTs), is up-regulated and piperlongumine directly interacts with GSTs and inhibits their activities. These results suggest that selective up-regulation of oxidative stress response enzymes can be a novel therapeutic target and piperlongumine represents a new class of chemotherapeutics.

In order to examine the contribution of ROS-induced DNA damage to the cytotoxicity of piperlongumine and the impact of a DNA repair pathway to the cytotoxicity of piperlongumine, a panel of DNA repair-deficient cell lines derived from chicken DT40 cells was studied. Our results show that piperlongumine selectively kills cell lines with a defect in homologous recombination (HR). Piperlongumine displays little or no toxicity to cell lines with a defect in other DNA repair pathways, including the base excision repair (BER) that is a major pathway to repair ROS-induced DNA lesions. A deletion of 53BP1 or Ku70 in BRCA1-deficient cell lines restores resistance to piperlongumine, strongly implicating that piperlongumine exerts its cytotoxicity by generating double strand breaks. Unexpectedly, we also discovered that piperlongumine suppresses HR. Altogether, we described the novel mechanisms of cytotoxicity by piperlongumine.

## RESULTS

### Cellular sensitivity profile of piperlongumine in a panel of isogenic DNA repair mutant DT40 cell lines

**Figure 1:**
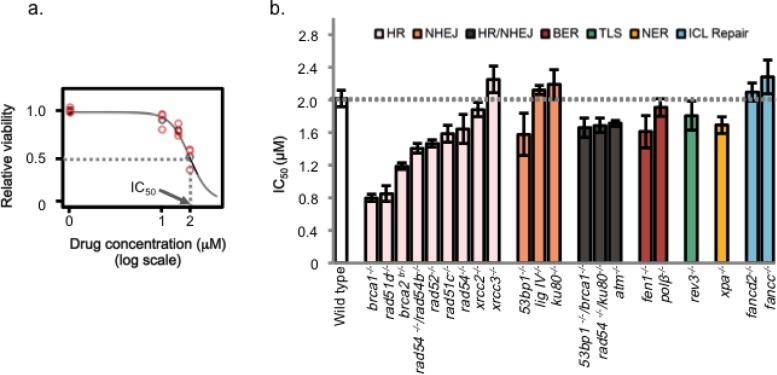
The cellular sensitivities of DNA repair deficient DT40 mutants to piperlongumine a. *Obtaining IC_50_ values of piperlongumine in each DNA repair –deficient cell-line.* In each experiment, the relative viabilities are measured as N/N_0_, where N is the number of colonies at each dose of piperlongumine-treated cells and N_0_ is the mean colony number of non-treated controls. The surviving fractions are marked with red symbols and the mean value at each dose is represented by a black open circle. b. *Piperlongumine specifically sensitizes HR-deficient DT 40 cells.* DNA repair deficient cells were treated with various doses of piperlongumine for 24 h and grew in the methylcellulose- containing medium for 7 days at 39°C. After the Giemsa staining, the numbers of colonies formed were counted and IC_50_ was determined. The representative dose response curves used to determine IC_50_ were shown in Supplementary Figure S1. All experiments were performed in triplicate. The IC_50_ values were plotted as dots and the SEMs were shown as error bars; and the dotted vertical line represents the IC_50_ of the wild type (2.0 μM). The order of DT40 mutants in the graph is based on their IC_50_ values.

Piperlongumine induces an elevated level of reactive oxygen species (ROS). To examine the contributions of ROS-induced DNA damage to the cytotoxicity of piperlongumine, the cellular sensitivity of various DNA repair-deficient, chicken DT40 mutant cell lines was investigated using colony formation assay. Due to the availability of isogenic DNA repair deficient cell lines, chicken DT40 cell lines have been used to study the mechanisms of DNA repair and genome instability [13, 14]. Various isogenic DNA repair-deficient DT40 cell lines (listed in Supplementary Table S1) were exposed to piperlongumine constantly and the cellular sensitivity of each mutant cell line was determined. IC_50_, that is a concentration of piperlongumine that killed the cell to the level of 50% of the control culture, was determined for each DNA repair-deficient mutant cell line (Figure 1b, Supplementary Figure S1). Only the HR-deficient cell line, including *brca1^−/−^* and *brca2^tr/−^* showed hyper-sensitivity to piperlongumine. These data suggest that piperlongumine induce DNA double strand breaks (DSBs).

DSBs can be generated directly by ROS and also by DNA interstrand cross-links and protein-DNA cross-links during replication. Repair of DNA interstrand cross-links and protein-DNA cross-links requires Fanconi anemia (FA), nucleotide excision repair (NER) genes and HR. Since FA-deficient cell lines, *fancc^−/−^* and *fancd2^−/−^*, and the NER-deficient cell line, *xpa^−/−^* are not sensitive to piperlongumine, we can eliminate DNA interstrand cross-links and protein-DNA cross-links as the cytotoxic lesions induced by piperlongumine. ROS-induced base modifications are mainly repaired by base excision repair (BER). Since the cell-line-deficient BER-related genes, *polβ^−/−^* and *fen1^−/−^,* did not show elevated sensitivity to piperlongumine, ROS-induced base modifications are not the cytotoxic lesions induced by piperlongumine (Figure 1b). We conclude that DSBs are the major cytotoxic lesions induced by piperlongumine.

**Figure 2:**
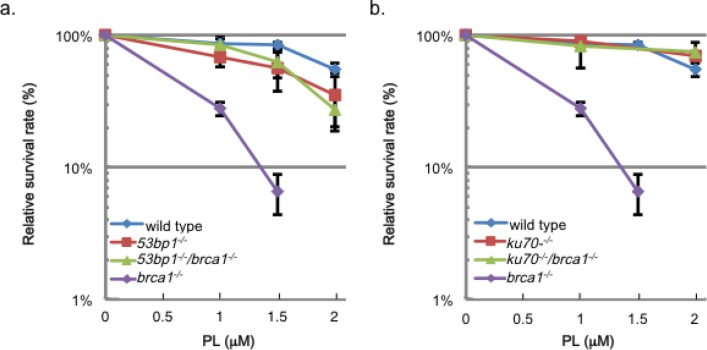
A deletion of 53BP1 or Ku70 abrogates the piperlongumine-induced cytotoxicity in *brca1^−/−^. DT40 cell-line* Each cell-line was treated with the indicated doses of piperlongumine (PL) and the surviving fractions were obtained. The error bars represent SEM obtained from three independent experiments. While a single deletion mutant of 53BP1 or Ku70 did not show sensitivity to PL, a deletion of 53BP1 or Ku70 in the *brca1^−/−^* cell-line abolished the cellular sensitivity of PL in this mutant cell-line. The two graphs were generated from the same set of experiments. In order to better demonstrate the results, the experiments with 53BP1 and the ones with Ku70 were graphed separately.

DSBs are repaired by HR and non-homologous end joining (NHEJ) [15]. Cells deficient in Ku80, LigIV and 53BP1 displayed resistance to piperlongumine (Figure 1b). Thus, NHEJ is not the major contributor for the repair of DSBs generated by piperlongumine. Recent studies demonstrate interplay and the competition of HR and NHEJ. In BRCA1- deficient mammalian cells, 53BP1 binds to DSBs and inhibits the end-resection process by MRN and CtIP, and promotes the initiation of NHEJ. Inactivation of 53BP1 in BRCA1-deficient cells restores viability/cell growth defect and the HR activity [16, 17]. This restoration of the HR activity alleviates cellular hyper-sensitivity and genomic instability (chromosomal aberrations) induced by DNA damaging agents, such as PARP- inhibitors and camptothecin in BRCA1-deficient cells. Analogous to these reports, a deletion of 53BP1 or Ku70 in the Brca1- deficient DT40 cell line restored the cellular resistance to piperlongumine (Figure 2). These results further support that DSBs are the major cytotoxic lesions induced by piperlongumine.

### Piperlongumine induces the recruitment of Rad51 to chromatin and causes chromosomal breakages in wild type DT40 cells

**Figure 3:**
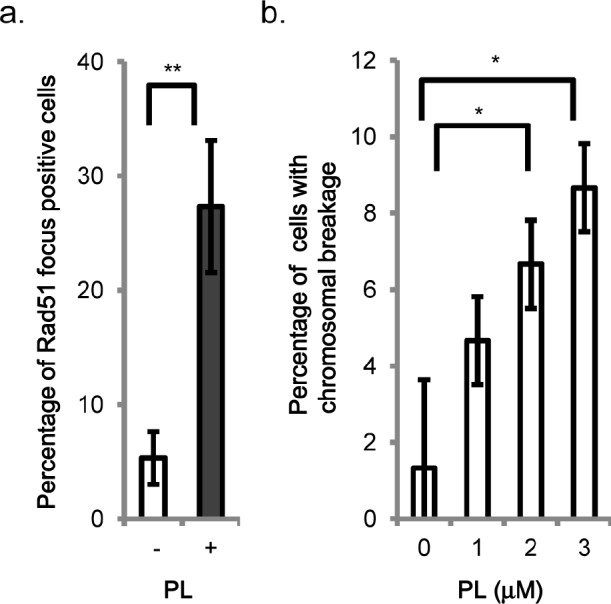
Evidence for piperlongumine-induced DNA damage a. *Induction of Rad51 foci by piperlongumine*. Wild type DT40 cells were stained with anti-RAD51 antibody after a 24 h exposure to 2 μM piperlongumine. Cells with more than three bright Rad51 foci were counted as positive. b. *Piperlongumine induces chromosomal aberrations*. Wild type DT40 cells were incubated for 24 h with indicated doses of piperlongumine. Number of chromosomal aberrations per 50 metaphase nuclei from the indicated cells was counted. Data are presented as mean ± SEM. * *p*-value<0.05, ** *p*-value<0.01.

The recruitment of the Rad51 protein to chromatin after the induction of DSBs is a critical step in HR [18]. To confirm that piperlongumine induces DSBs, the recruitment of Rad51 to chromatin was examined by immunofluorescence assay. As shown in Figure 3a, the formation of Rad51 foci was detected 24 h after the piperlongumine treatment in wild type DT40 cells (Supplementary Figure S2), indicating the formation of DSBs.

DSBs, if not repaired properly, will result in chromosome breakages. Asynchronous cultured cells were exposed to a different concentration of piperlongumine. After 24 h of incubation, mitotic cells were harvested, and chromosomal breakages were measured in wild type cells [18]. As shown in Figure 3b, piperlongumine induced chromosomal breakages and the numbers of chromosomal breakages were elevated in a concentration- dependent manner. The results show that piperlongumine-induced DSBs result in chromosome breakages.

### Piperlongumine suppresses homologous recombination

**Figure 4:**
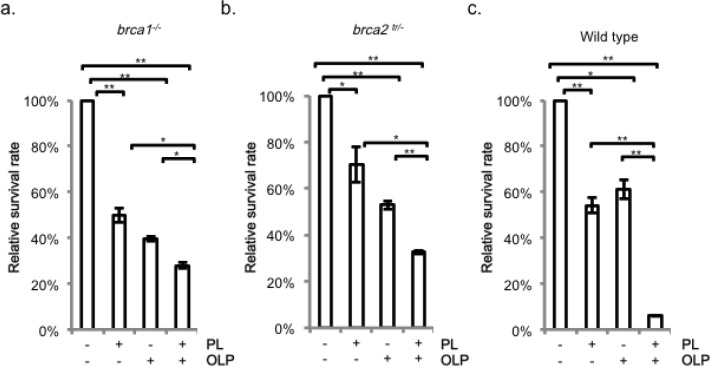
Effect of PARP-inhibitor olaparib on the cellular sensitivity to piperlongumine in *brca1^−/−^* and *brca2tr/−* cell-lines Cellular sensitivity of piperlongumine was investigated in the presence and absence of olaparib. Cells were treated with the indicated combinations and doses of chemicals for 24 hr and, after washing the drugs, the cells were grown for 7 days. a. *brca1^−/−^.*, b. *brca2^tr/−^* and c. wild type. Piperlongumine was added at 1 μM and olaparib was at 25 nM in a and b, while piperlongumine was at 2 μM and olaparib was at 5 μM in c. Data were presented as mean ± SEM. * *p*-value<0.05, ** *p*-value<0.01.

Our genotoxic analyses with DT40 DNA repair-deficient cell lines show that piperlongumine induces DSBs. Next, we wished to examine the impact of PARP- inhibitors on the cytotoxicity of piperlongumine. Inhibition of poly (ADP-ribose) polymerase 1 (PARP1) selectively sensitizes HR-deficient cells, including Brca1- deficient and BRCA2-deficient cells [19-21]. It is believed that DSBs generated from the accumulated SSBs during replication by the inhibition of PRAP1 selectively kill HR- deficient cells [22]. Interestingly, the PARP inhibitor, olaparib (AZD-2281), sensitized *brca1^−/−^* and *brca2tr/−* to piperlongumine moderately (Figures 4a and b). Surprisingly, in sharp contrast to HR-deficient cell lines, olaparib significantly enhanced the cytotoxicity of piperlongumine in wild type cells (Figure 4c). The results strongly implicate that piperlongumine suppresses HR.

**Figure 5:**
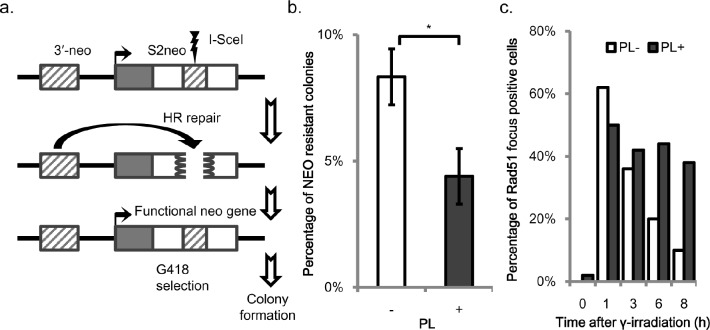
Suppression of homologous recombination by piperlongumine a. *Cell-based homologous recombination (HR) assay in DT40*. The expression vector encoding I-SceI is transfected to cells with *SCneo* (*3′neo* and *S2neo*) in the *Ovalbumin* locus. Black and white box represent 5′-untranslated region and coding regions of the *neo^R^* gene, respectively. The figure is not drawn to the scale. b. *Pieprlongumine suppresses HR in DT40.* Immediately after wild type DT40 cells were transfected with I- SceI expression vector, the cells were grown for 48 h in the RPMI medium in the presence and absence of 1 μM of piperlongumine. Then, the cells were diluted appropriately, seeded, and grown in the presence of 2 mg/ml G418 in 96-well plates. The recombination frequency was calculated by dividing the number of neomycin-resistant colonies by the number of the total colonies. Data are presented as mean ±SEM. * *p*-value<0.05. c. *Impact of piperlongumine on the* γ*-ray-induced Rad51 foci formation.* Wild type DT40 cells were treated with piperlongumine (PL) at 1 μM for 1 h. After removing piperlongumine, the cells were irradiated with γ-ray at 2 Gy. Foci-formations of Rad51 were examined at the indicated time points after the irradiation. Irradiated wild type cells without treatment with piperlongumine were used as a control. A cell containing more than three distinct foci was scored as positive. Each bar represents the results of scoring at least 50 cells. Data are presented as mean ±SEM.

To investigate the impact of piperlongumine on HR directly, a cell-based HR assay was performed. An *SCneo* reporter gene with a restriction enzyme I-SceI cutting site was inserted at the *Ovalumin* locus [23]. This SCneo reporter gene includes two mutant neo-resistance genes, *SCEneo* and *3'-neo*, localized in tandem [23] (Figure 5a). One neo region (*SCEneo*) was disrupted followed by the transient expression of I-SceI and the induction of a DSB. A functional neomycin-resistant gene is restored only when the disrupted *SCEneo* is repaired by HR using the *3'-neo* gene as a donor. Therefore, HR activity can be evaluated by counting neomycin-resistant colonies followed by I-SceI transient expression. The number of G418-resistant colonies was reduced by 50% in wild type cells by the treatment with piperlongumine (Figure 5b, Supplementary Figure S2).

To obtain insight into the mechanism of the suppression of HR by piperlongumine, we investigated the kinetics of Rad51 accumulation after γ-ray irradiation (Figure 5c, Supplementary Figure S3). The number of Rad51 foci-positive cells was counted at each time point after the irradiation. The initial recruitment of Rad51 after γ-ray irradiation was delayed with the piperlongumine treatment. Numbers of γ-ray induced Rad51 foci were decreased with time in the absence of piperlongumine, indicating the completion of the repair of DSBs. In contrast, γ-ray induced Rad51 foci were sustained even 8 h after incubation in the presence of piperlongumine (Figure 5c, Supplementary Figure S3).

These data demonstrate that piperlongumine induces DSBs and also suppresses HR.

## DISCUSSION

Recently, it was demonstrated that piperlongumine increased the level of reactive oxygen species (ROS) and apoptotic cell death selectively in various types of cancer cells with minimal cytotoxicity to non-transformed cells [11]. In the same study, they clearly showed that the elevation of the ROS level is due to the inhibition of the ROS response enzyme, glutathione S-transferases (GSTs) by direct interaction with GSTs [11]. In this study, we investigated the contribution of DNA repair pathways to the cytotoxicity of piperlongumine. We performed a comprehensive genetic analysis with multiple DNA repair pathways in response to piperlongumine using various DNA repair-deficient chicken DT40 cell lines. This report is the first to compare the cellular sensitivity of different DNA repair-deficient cell lines to piperlongumine quantitatively. Sensitivity profiles (IC_50_) of piperlongumine in various DNA repair-deficient cell lines showed that HR repair-deficient cell lines display a higher cellular sensitivity to piperlongumine compared with any other DNA repair-deficient cell lines (Figure 1, Supplementary Figure S1). These observations strongly suggest that the major piperlongumine-induced cytotoxic DNA damage is DSBs. The presence of DSBs was confirmed by Rad51 accumulation in chromatin (Figure 3a, Supplementary Figure S2) and chromosome breakage by the treatment with piperlongumine (Figure 3b). DSBs are generated directly and indirectly by various DNA-damaging agents. SSBs, DNA interstrand cross-links, and protein-DNA cross-links can induce DSBs during replication. Our genetic experiments with various DNA repair-deficient cell lines exclude the possibilities that the cytotoxicity of piperlongumine is due to the formation of DNA interstrand cross-links and protein-DNA cross-links as well as bulky DNA lesions. The source of DSBs and/or SSBs in piperlongumine-treated cells is currently under investigation.

It is very interesting that *brca1^−/−^* cells exhibit a higher cellular sensitivity to piperlongumine compared to other HR repair-deficient cells, including *brca2tr/−*. BRCA1 is known to possess various functions outside of a role in HR. It was reported that BRCA1 plays a yet unidentified role in the repair of DNA interstrand cross-links in mammalian cells [24]. It is also noted that mammalian BRCA1 transcriptionally regulates BER proteins such as 8-oxoguanine DNA glycosylase (OGG1) [25]. The DNA glycosylase, NTH1 and the apurinic endonuclease 1 (APE1) and BRCA1-deficient cells are reportedly sensitive to ROS-induced oxidative DNA damage [26, 27]. Due to dysfunctional BER in the BRCA1-deficient cell line, oxidative DNA damage induced by piperlongumine might be processed to SSBs and then converted to DSBs during replication. These additionally generated DSBs might explain a higher cytotoxicity of piperlongumine in BRCA1-deficient cell lines compared to other HR-deficient cell lines that retain intact BER activity.

Unexpectedly, we observed significantly enhanced cytotoxicity in wild type DT40 cells by a combination treatment of piperlongumine with olaparib (Figure 4c). Further analysis with the cell-based HR assay and a kinetic study of Rad51-foci formation clearly demonstrate that piperlongumine suppresses the HR activity (Figure 5). Piperlongumine delays the recruitment of Rad51 to chromatin damage by γ-ray (Figure 5c, Supplementary Figure S3), suggesting that the suppression is temporal and targets a process upstream of Rad51; however, a mechanistic basis of the suppression of HR by piperlongumine remains elusive. We are currently investigating what stage of the HR process is compromised by piperlongumine.

In summary, we revealed two novel activities of piperlongumine. One is the induction of DSBs and the other is the suppression of HR. These findings make piperlongumine the more attractive candidate for chemotherapy of breast and ovarian cancers with defective HR. Our results also indicate that piperlongumine can be used against PARP-inhibitor- resistant, BRCA1-deficient cancers.

## MATERIALS AND METHODS

### Cell lines and cell culture

The DT40 cell lines used in this study were generated in the Laboratory of Radiation Genetics, Graduate School of Medicine, Kyoto University (Kyoto, Japan). All the mutant cell lines were previously authenticated by Southern blotting, PCR and/or Western blotting (Supplementary Table S1). All DNA repair-deficient, mutant cell lines are isogenic to the wild type cell line.

Both wild type and mutant DT40 cells were cultured at 39°C with 5% CO2 by using RPMI 1640 medium supplemented 10% fetal bovine serum, 1% chicken serum, 100 U/ml penicillin, and 100 U/ml streptomycin, 50 μM β -mercaptoethanol, and 2 mM L – glutamine [18].

### Measurement of cytotoxicity to chemicals

Colony formation assay was described previously [28]. Briefly, serially diluted cells were plated in triplicated 60-mm dishes with 8 ml of DMEM/F-12 containing 1.5% methylcellulose, 2 mM L-glutamine, 15% of FCS, and 1.5% of chicken serum, with or without different concentrations of piperlongumine (BioVision). For combination drug experiments, cells were incubated for 24 h in complete RPMI 1640 medium with or without an appropriate concentration of the drug. After 24 h of incubation, serially diluted cells were plated in triplicated methylcellulose containing DMEM/F112 medium as described previously.

In each experiment, colonies were counted after 7 days of incubation at 39 °C. the relative viabilities are measured as N/N_0_, where N is the number of colonies at each dose of piperlongumine-treated cells and N_0_ is the mean colony number of non-treated controls. The survival curves were obtained with a three-parameter logistic curve using package dose response curve in R [29]. The representative dose response curves used to determine the IC_50_ were shown in Supplementary Figure S1.

### Rad51 foci formation analysis

To visualize sub-nuclear foci formation of Rad51 in DT40 cells, cells were harvested by using Cytospin (SHANDON). Staining and visualization of Rad51 foci were performed using RAD51 antibody (Bioacademia, 11-536) as previously described [30]. Cells with more than three brightly fluorescing foci were counted as positive. At least 100 morphologically intact cells were counted at each time point.

### Chromosome aberration assay

Analysis of chromosomal aberrations was performed as described previously [18]. Briefly, cells were treated for 3 h with medium containing 0.1 μg/ml Colcemid (Gibco). Harvested cells were incubated in 1 ml of 75 mM KCl for 15 min at room temperature and fixed in 5 ml of a freshly prepared 3:1 mixture of methanol-acetic acid. The cell suspension was dropped onto a slide and the slides were dried. The slides were stained with 5% Giemsa solution (pH 6.4) for 8 min. Data are presented as macro chromosomal aberrations per 50 meta-phase spreads [18].

### Cell-based homologous recombination (HR) assay

Modified *SCneo* [31] was inserted into the previously described *Ovalbumin* gene construct and targeted into the *Ovalbumin* locus in wild type cells [23]. I-SceI expression vector was transfected transiently into cells by electroporation using the Amaxa Nucleofector. After incubation with or without 1 μM piperlongumine for 48 h, cells were selected in 96-well culture plates containing 2 mg/ml G418 (Nacalai Tesque). Only cells with successful homologous recombination at the *SCneo* locus after the introduction of a DSB by I-SceI grow in the presence of G418.

### Statistical analysis

Three independent experiments were performed with each data set in this report unless stated otherwise. The results were expressed as mean ± SEM. Differences among the data were tested for statistical significance using the *t* test. P-values were determined using the *t* test.

## SUPPLEMENTARY FIGURES, TABLES AND REFERENCES


