# Supplementary material for: Impact of DNA repair pathways on the cytotoxicity of piperlongumine in chicken DT40 cell-lines
Source: Genes Cancer. 2014 Jul;5(7-8):285–92. doi: 10.18632/genesandcancer.26 (PMC4162141; doi:10.18632/genesandcancer.26)
Supplement: Supplementary file 1 [file ganc-05-285-s001.pdf]

Impact of DNA repair pathways on the cytotoxicity of piperlongumine in chicken DT40 cell-lines.

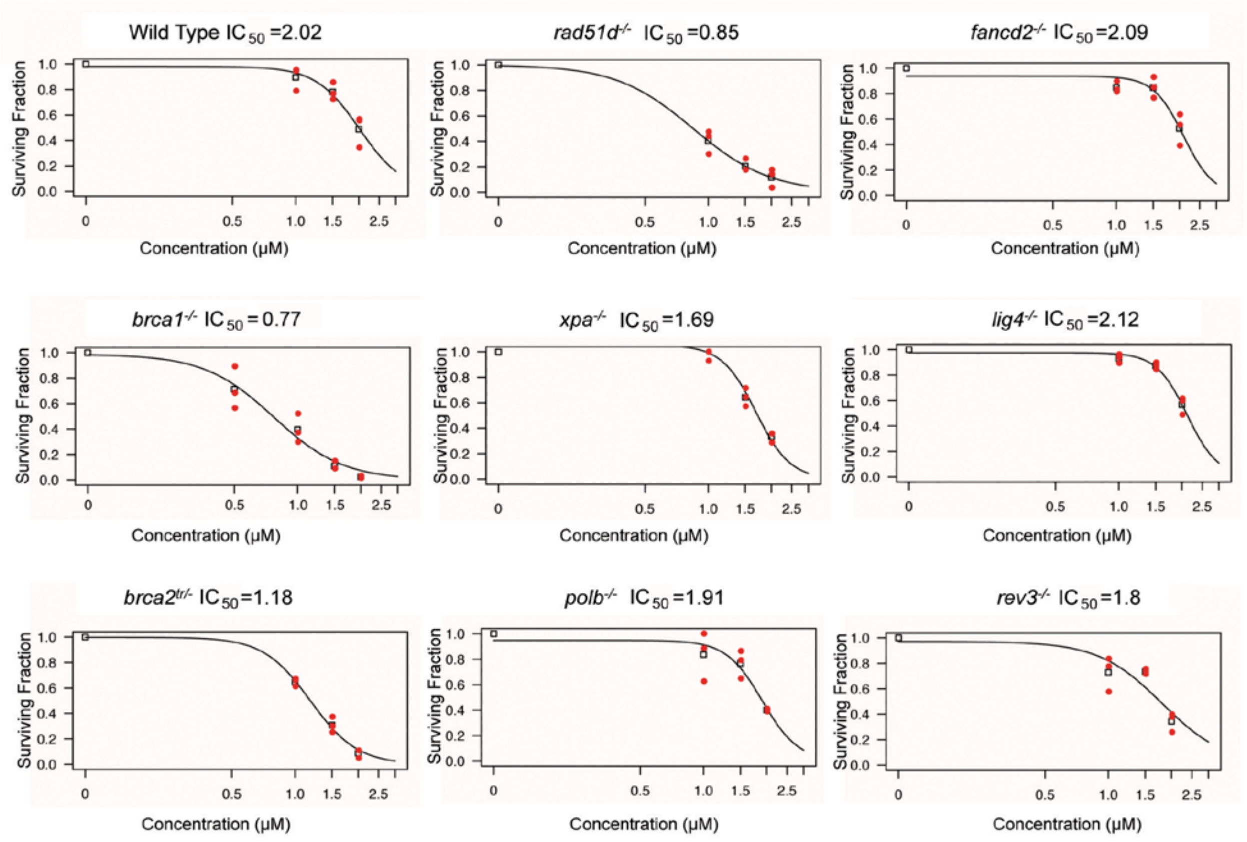

Supplementary Figure S1. *Dose response curves to piperlongumine in representative DNA repair deficient DT40 cell-lines.*

Each cell-line was exposed to the indicated concentrations of piperlongumine and the surviving fractions were calculated. The red dots represent surviving fraction at each concentration of piperlongumine and the open squares represent mean values at each concentration. The dose response curves (survival curves) were obtained with a three- parameter logistic curve using package dose response curve in R.

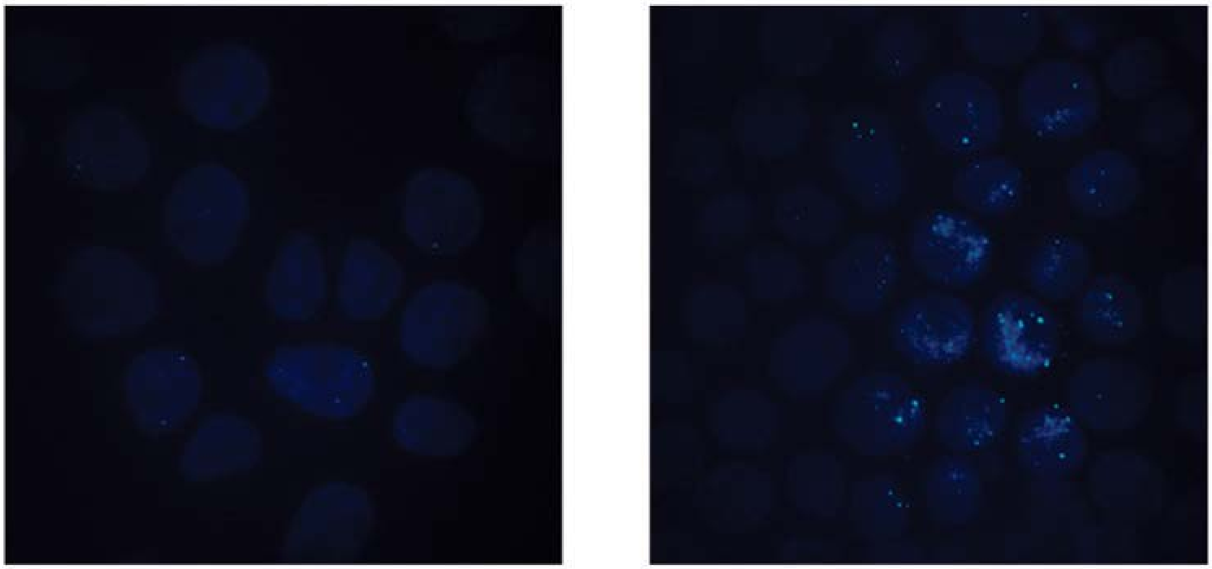

Supplementary Figure S2. *Induction of Rad51-foci formation by piperlongumine*. Wild type DT40 cells were treated with 2 μM of piperlongumine (PL) for 24 hr. Representative images of control cells (left panel) and PL-treated cells (right panel) were shown.

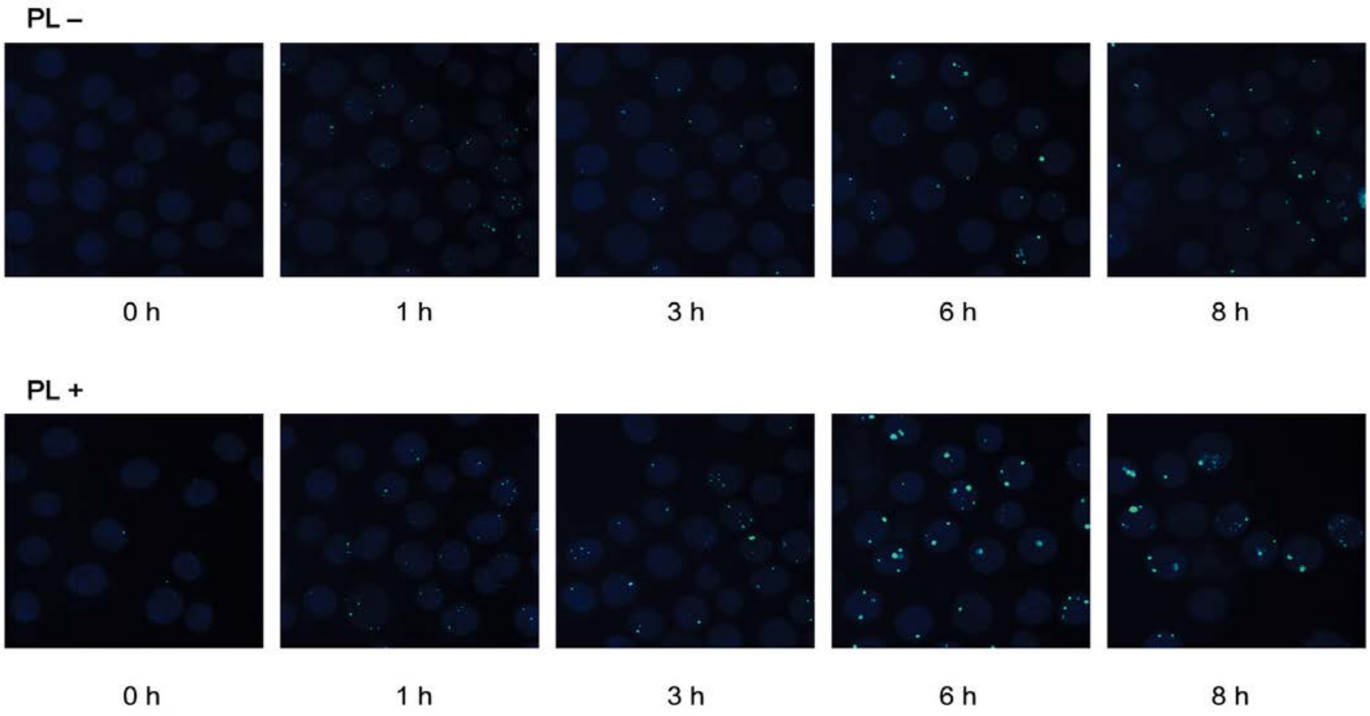

Supplementary Figure S3. *Suppression of homologous recombination by piperlongumine*.

Wild type DT40 cells were pre-treated with 1 μM of piperlongumine (PL) for 1 hr. After removing piperlongumine, the cells were irradiated with γ-ray at 2 Gy. Foci-formations of Rad51 were examined at the indicated time points after the irradiation. The upper panel: Irradiated cells without pre-treatment with PL. The lower panel: Irradiated wild type cells with pre-treatment with PL Representative images were shown.

Supplementary Table S1
DT40 isogenic DNA repair mutant cells used in this study.

Cell line	Function inactivated	References
ku70$^{-/-}$	NHEJ	(Takata et al., 1998)
ligase IV$^{-/-}$	NHEJ	(Adachi et al., 2001)
53bp1$^{-/-}$	Inhibition of HR	(Nakamura et al., 2006)
atm$^{-/-}$	DDR	(Takao et al., 1999)
rad52$^{-/-}$	HR, SSA	(Yamaguchi-Iwai et al., 1998)
xrcc2$^{-/-}$	HR	(Takata et al., 2001)
xrcc3$^{-/-}$	HR	(Takata et al., 2001)
brca1$^{-/-}$	HR	(Martin et al., 2007)
brca2$^{tr/-}$	HR	(Hatanaka et al., 2005)
fancc$^{-/-}$	ICL	(Hirano et al., 2005)
fancd2$^{-/-}$	ICL	(Yamamoto et al., 2005)
polb$^{-/-}$	BER	(Tano et al., 2007)
xpa$^{-/-}$	NER	(Okada et al., 2002)
rev3$^{-/-}$	TLS	(Sonoda et al., 2003)
rad51c$^{-/-}$	HR	(Takata et al., 2001)
rad54$^{-/-}$ *ku80*$^{-/-}$	HR, NHEJ	(Takata et al., 1998)
fen1$^{-/-}$	BER	(Matsuzaki et al., 2002)
53bp1$^{-/-}$*brca1*$^{-/-}$		Unpublished
rad54$^{-/-}$*rad54b*$^{-/-}$	HR	Unpublished

References

Adachi, N., Ishino, T., Ishii, Y., Takeda, S., and Koyama, H. (2001). DNA ligase IV-deficient cells are more resistant to ionizing radiation in the absence of Ku70: Implications for DNA double-strand break repair. Proc Natl Acad Sci U S A *98*, 12109-12113.

Hatanaka, A., Yamazoe, M., Sale, J.E., Takata, M., Yamamoto, K., Kitao, H., Sonoda, E., Kikuchi, K., Yonetani, Y., and Takeda, S. (2005). Similar effects of Brca2 truncation and Rad51 paralog deficiency on immunoglobulin V gene diversification in DT40 cells support an early role for Rad51 paralogs in homologous recombination. Mol Cell Biol *25*, 1124-1134.

Hirano, S., Yamamoto, K., Ishiai, M., Yamazoe, M., Seki, M., Matsushita, N., Ohzeki, M., Yamashita, Y.M., Arakawa, H., Buerstedde, J.M., *et al.* (2005). Functional relationships of FANCC to homologous recombination, translesion synthesis, and BLM. EMBO J *24*, 418-427.

Martin, R.W., Orelli, B.J., Yamazoe, M., Minn, A.J., Takeda, S., and Bishop, D.K. (2007). RAD51 up-regulation bypasses BRCA1 function and is a common feature of BRCA1-deficient breast tumors. Cancer Res *67*, 9658-9665.

Matsuzaki, Y., Adachi, N., and Koyama, H. (2002). Vertebrate cells lacking FEN-1 endonuclease are viable but hypersensitive to methylating agents and H2O2. Nucleic Acids Res *30*, 3273-3277.

Nakamura, K., Sakai, W., Kawamoto, T., Bree, R.T., Lowndes, N.F., Takeda, S., and Taniguchi, Y. (2006). Genetic dissection of vertebrate 53BP1: a major role in non-homologous end joining of DNA double strand breaks. DNA Repair (Amst) *5*, 741-749.

Okada, T., Sonoda, E., Yamashita, Y.M., Koyoshi, S., Tateishi, S., Yamaizumi, M., Takata, M., Ogawa, O., and Takeda, S. (2002). Involvement of vertebrate polkappa in Rad18-independent postreplication repair of UV damage. J Biol Chem *277*, 48690-48695.

Sonoda, E., Okada, T., Zhao, G.Y., Tateishi, S., Araki, K., Yamaizumi, M., Yagi, T., Verkaik, N.S., van Gent, D.C., Takata, M., *et al.* (2003). Multiple roles of Rev3, the catalytic subunit of polzeta in maintaining genome stability in vertebrates. EMBO J *22*, 3188-3197.

Takao, N., Kato, H., Mori, R., Morrison, C., Sonada, E., Sun, X., Shimizu, H., Yoshioka, K., Takeda, S., and Yamamoto, K. (1999). Disruption of ATM in p53-null cells causes multiple functional abnormalities in cellular response to ionizing radiation. Oncogene *18*, 7002-7009.

Takata, M., Sasaki, M.S., Sonoda, E., Morrison, C., Hashimoto, M., Utsumi, H., Yamaguchi-Iwai, Y., Shinohara, A., and Takeda, S. (1998). Homologous recombination and non-homologous end-joining pathways of DNA double-strand break repair have overlapping roles in the maintenance of chromosomal integrity in vertebrate cells. EMBO J *17*, 5497-5508.

Takata, M., Sasaki, M.S., Tachiiri, S., Fukushima, T., Sonoda, E., Schild, D., Thompson, L.H., and Takeda, S. (2001). Chromosome instability and defective recombinational repair in knockout mutants of the five Rad51 paralogs. Mol Cell Biol *21*, 2858-2866.

Tano, K., Nakamura, J., Asagoshi, K., Arakawa, H., Sonoda, E., Braithwaite, E.K., Prasad, R., Buerstedde, J.M., Takeda, S., Watanabe, M.*, et al.* (2007). Interplay between DNA polymerases beta and lambda in repair of oxidation DNA damage in chicken DT40 cells. DNA Repair (Amst) *6*, 869-875.

Yamaguchi-Iwai, Y., Sonoda, E., Buerstedde, J.M., Bezzubova, O., Morrison, C., Takata, M., Shinohara, A., and Takeda, S. (1998). Homologous recombination, but not DNA repair, is reduced in vertebrate cells deficient in RAD52. Mol Cell Biol *18*, 6430-6435.

Yamamoto, K., Hirano, S., Ishiai, M., Morishima, K., Kitao, H., Namikoshi, K., Kimura, M., Matsushita, N., Arakawa, H., Buerstedde, J.M.*, et al.* (2005). Fanconi anemia protein FANCD2 promotes immunoglobulin gene conversion and DNA repair through a mechanism related to homologous recombination. Mol Cell Biol *25*, 34-43.
